# Advances in Computer Recognition, Image Processing and Communications

**DOI:** 10.3390/e24010108

**Published:** 2022-01-10

**Authors:** Michał Choraś, Robert Burduk, Agata Giełczyk, Rafał Kozik, Tomasz Marciniak

**Affiliations:** 1 Faculty of Telecommunications, Computer Science and Electrical Engineering, Bydgoszcz University of Science and Technology, 85-796 Bydgoszcz, Poland; agata.gielczyk@pbs.edu.pl (A.G.); rkozik@pbs.edu.pl (R.K.); tommar@pbs.edu.pl (T.M.); 2 Department of Systems and Computer Networks, Wroclaw University of Science and Technology, 50-370 Wroclaw, Poland; robert.burduk@pwr.edu.pl

This Special Issue aimed to gather high-quality advancements in theoretical and practical aspects of computer recognition, pattern recognition, image processing and machine learning (shallow and deep), including, in particular, novel implementations of these techniques in the areas of modern telecommunications and cybersecurity. As almost all human activities have been moved online due to the pandemic, novel robust and efficient approaches and further research have been in higher demand in the field of computer science and telecommunication.

The selected authors of the contributions presented on 12th International Conference on Computer Recognition Systems (CORES) and the 12th International Conference on Image Processing and Communications (IP&C), held jointly with the 22nd International Conference on Advanced Computer Systems (ACS), were invited to submit extended versions of their original papers for this Special Issue. Our multi-conference took place in June 2021. This Special Issue was open to other submissions outside of the conference, too.

Finally, after a rigorous review process, we accepted 13 interesting papers in various domains and applications of computer recognition, image processing and communications.

Image processing can be applied in numerous areas of our daily lives: for entertainment, security and also improving medicine. This type of computer vision implementation was presented in [1]. In this paper, the authors proposed a method for detecting veins. They used digital image correlation (DIC) for detecting the micro-shifts in the skin caused by pulsation of the underlying veins. This method enabled observing the thickness of the veins up to a certain length. This also allowed for an initial, quick determination of whether the patient should be referred for diagnostic tests for venous embolism and thrombosis.

In [2], the authors investigated whether it is possible to use data pre-processing methods to robustify an ANN-based classifier against an adversarial evasion attack in the person re-identification problem. They examined the set of following methods: JPEG compression, Gaussian noise, Local Spatial Smoothing, Total Variance Minimisation and Block-Matching Convolutional Neural Network (BMCNN) for image denoising. Furthermore, they proposed a pre-processing pipeline that can robustly defend the ANN-based classifier against the adversarial attack without re-training the classifier. The proposed method is especially valuable since in the computer vision domain, the training process can be a huge computational endeavor.

The authors in [3] focused on the problem of quality assessment of stitched images. The process of stitching is an important element of many virtual reality and remote sensing applications where the panoramic images may be used as a background. As it is a complex issue (stitching quality can be affected by geometric distortions, ghosting, blurring and color distortion), the authors aimed to develop a new objective image quality metric based on the entropy measure. The metric proposed in the paper ensured achieving a considerably higher correlation of the designed objective metrics with subjective quality scores of the stitched images delivered in the ISIQA database, which was used in the research.

In the article [4], a novel approach to pointer instrument reading was presented. As the digitalization of the industry has become widely implemented, the automatization of instrument reading has become an emerging issue. The authors proposed the following pipeline: instrument detection from an image (VGG-16), pointer position extraction (Hough transform) and, finally, the pointer position reading (analysis of the angle between the center line and the extracted pointer). The presented experiments proved that the proposed method can read the pointer instruments comparably to human vision (1.354% of relative error).

The papers [5,6] addressed different problems of modeling a smooth monotone curve. Modeling is an effective tool for investigating objects, phenomena and processes. It can also be applied in reverse engineering.

In [7], a novel approach to remote sensing image segmentation is proposed. The authors implemented their method in order to distinguish the crop and vacancy fields on the farmland images obtained by UAV. In this approach, the ResNet architecture was analyzed, improved and then implemented as a part of the encoder–decoder structure. The authors also claimed that the proposed method can be easily adapted to handle other similar issues, e.g., crack segmentation.

In [8], the problem of selecting an optical channel for transporting the double sideband radio-frequency-over-fiber (DSB-RFoF) radio signal over the optical fronthaul path, avoiding the dispersion-induced power penalty (DIPP) phenomenon, is addressed. The presented method complements the possibilities of a short-range optical network working in the flexible dense wavelength division multiplexing (DWDM) format, where chromatic dispersion compensation is not applied. In [9], the authors describe their algorithm for dynamic routing with dedicated path protection. They proposed the algorithm in the context of optical networks, but it can be applicable to other networks, where services have to be protected and the network resources are finite and discrete, e.g., wireless radio or networks capable of advance resource reservation.

In [10], the authors presented a deep neural network in order to automatically classify ECG signals. Three neural network architectures were proposed: the first based on the convolutional network, the second on SincNet and the third on the convolutional network but with additional entropy-based features. The author of [11] touches on a new approach in the last-mile network structural solutions for smart grid networks and suggests a new method for finding the optimal SM localization, which can also work as a data concentrator.

In [12], the authors proposed Hfinger, a novel effective malware HTTP request fingerprinting tool. It extracts information from the parts of the request, such as URI, protocol information, headers and payload, providing a concise request representation that preserves the extracted information in a form interpretable by a human analyst.

The authors of [13] establish the minimal amount of data that is sufficient to efficiently train machine learning algorithms in intrusion detection. The authors also identify the most valuable NetFlow-based features that facilitate effective, machine-learning-based network intrusion detection in the real world. Their objectives are reached in a series of experiments with the use of several feature selection techniques, machine learning algorithms and intrusion detection benchmark datasets. The paper [13] is the result of the EU Horizon 2020 SIMARGL project (simargl.eu).
